# Commemorating the monkey bars, catalyst of debate at the intersection of human evolutionary biology and public health

**DOI:** 10.1093/emph/eoae017

**Published:** 2024-08-19

**Authors:** Luke D Fannin, Zaneta M Thayer, Nathaniel J Dominy

**Affiliations:** Department of Anthropology, Dartmouth College, Hanover, NH, USA; Graduate Program in Ecology, Evolution, Environment, and Society, Dartmouth College, Hanover, NH, USA; Department of Anthropology, Dartmouth College, Hanover, NH, USA; Department of Anthropology, Dartmouth College, Hanover, NH, USA; Department of Biological Sciences, Dartmouth College, Hanover, NH, USA

**Keywords:** risky play, playground equipment, pediatric bone fracture, anxiety

## Abstract

Play is an essential part of childhood, and growing attention has focused on the potential health benefits of ‘risky’ or ‘thrill-seeking’ play. Such play behavior is readily observed on any playground, where it can sometimes lead to injuries––most often from fall impacts––that require medical attention. Monkey bars account for ~7% of childhood arm fractures in the USA, an alarming statistic that raises difficult questions over its costs and benefits. Many authors view monkey bars as a public health hazard, but it is plausible that our childhood impulse toward thrill-seeking play is a result of selective pressures throughout our primate evolutionary history. Indeed, emerging evidence suggests that the developmental benefits of thrill-seeking play extend into adulthood, outweighing the occasional costs of injury. Disparate and consequential, these dueling perspectives have fueled debate among health professionals and policymakers, but with little attention to the work of biological anthropologists. Here we call attention to the hominin fossil record and play behaviors of non-human primates, providing a novel perspective that bolsters arguments for the adaptive significance of thrill-seeking play. The moment for such a review is timely, for it commemorates the centennial anniversaries of two playground icons: the jungle gym and monkey bars.

## INTRODUCTION

Hazards and risks are similar but distinct concepts. Hazards are potential sources of harm, whereas risks reflect the probability of harm [1]. It follows that risk-taking requires agency––a conscious decision to engage in activities with uncertain outcomes [2, 3]. Because its calculated nature demands physical and emotional management, two essential life skills [3], many developmental psychologists view risky play as integral to child growth and well-being, as children typically develop risk-management skills through such experiences [3]. However, the subtle distinction between hazard and risk is often blurred on modern playgrounds. Childhood injuries from ‘risky’, ‘adventurous’, or ‘thrill-seeking’ play in these settings has fueled parental anxiety and governmental regulation [4, 5]. It is plausible that risk mitigation efforts, although well-intentioned, have inadvertently harmed children’s psychological and physical development [6–8]. This debate over the costs and benefits of thrill-seeking play has far-reaching importance for child well-being and public health policy, but it is seldom addressed through the lens of human evolutionary biology, a discipline that is well-positioned to contextualize thrill-seeking play within a broader understanding of primate motor development.

**Figure 1.**
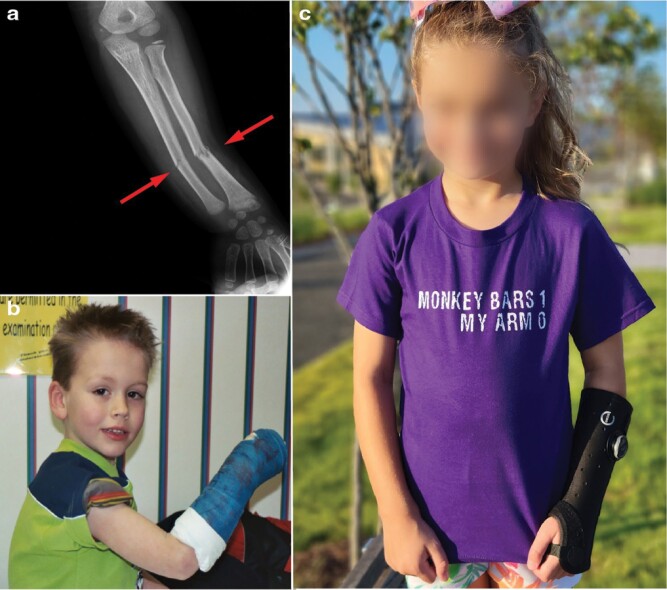
Playground falls and forelimb fractures. (a) Angular fractures (arrows) of the radial and ulnar diaphyses of a 6-year-old male. The radial fracture is complete and involves both cortices; however, the ulnar fracture is incomplete with cortical and buckle fractures, exemplifying so-called greenstick fractures. Greenstick fractures are common among children <10 years of age when an angulated longitudinal force is applied along the bone of an outstretched arm. Case courtesy of Samir Benoudina, Radiopaedia.org, rID: 21674. (b) L.D.F. at six years old in 2002, when radio-ulnar fractures from falls were treated with hard casts; photograph by Steve Fannin, reproduced with permission. (c) Today, most radio-ulnar fractures are treated with soft splints; photograph by Jennifer Bernstein, reproduced with permission.

Public perceptions of playground hazards are rooted in some alarming statistics. Monkey bars [Box 1], for instance, are responsible for more hospital visits and fractures during childhood than any other playground structure [9–12]. Most fractures are the result of fall impacts [13, 14], with heights of 2 m having an odds of fracture eleven times greater than those <1 m [15]. In Canada, monkey bars accounted for 5% of emergency department (ED) visits by children, 64% of which entailed bone fracture [10]. Another study found that monkey bars accounted for 50% of playground-related extremity fractures admitted to EDs in the USA, and 55% of severe extremity fractures [12]. This same study found strong cohort differences, with the greatest incidence of fracture occurring among 5–9 year-olds. Fractures of the wrist or forearm are most common (Fig. 1) [16], accounting for 6–21% of all bone fractures during childhood [17–19]. Rates of bone fracture are comparable between boys and girls [9], but a statistical difference emerges with monkey bars––girls are moderately more likely to suffer a fracture [9] despite similar levels of thrill-seeking play at ‘great heights’ [20]. Such sobering statistics prompted calls to reduce the height of monkey bars to <2 m, while also requiring deeper (~20–30 cm) and/or more compliant surface materials such as sand, bark chips or rubber [21–24].

**Box 1: Evolution and the monkey bars**

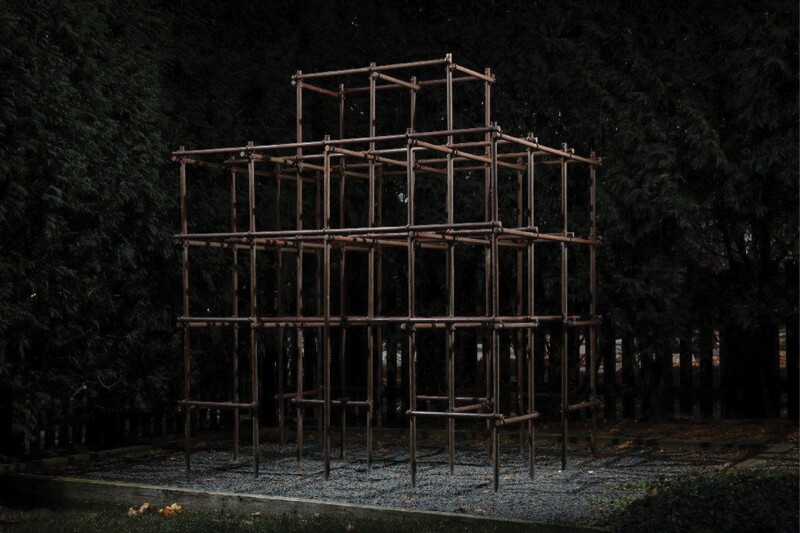

Original jungle gym at the Winnetka Historical Society, Winnetka, Illinois. Photograph by E. Jason Wambsgans. © Chicago Tribune/Tribune News Service, reproduced with permission.

The jungle gym was invented in 1920 by Sebastian (‘Ted’) Hinton, an attorney [116]. In a series of four patent applications––the first of which was awarded October 23, 1923––Hinton [117] described a metal climbing frame of layered cubes, eight feet tall, arranged like a ‘forest top through which a troop of children may play in a manner somewhat similar to that of a troop of monkeys through the treetops in a jungle’ (p. 1). His final application, approved in 1924, included an accessory monkey runway, which he described as an elevated horizontal ladder attached to the side of the jungle gym and suspended at the corners by vertical poles [118]. He argued that such a runway would ‘permit suspensory exercise, such as swinging by the arms and hanging travel, hand over hand’ (p. 2). Thus, Hinton’s climbing frame (today’s jungle gym) and monkey runway (today’s monkey bars) were envisioned as conjoined structures. Here, we view them interchangeable forms of playground equipment because both invite climbing, putting them into the same category of risky play––danger or injury from falling [3]. Most studies consider them together when reporting injuries [14].

Hinton lived in Winnetka, Illinois, a Chicago suburb and epicenter for the rise and spread of progressive education [116]. This factor was a major contributor to the success of his inventions. Winnetka school administrators, chief among them superintendent Carleton Washburn, were disciples of John Dewey, a prominent philosopher and proponent of ‘whole pupil’ education [119]. Dewey advocated for curricula that balanced formal academics with practical activities meant to promote the mental, physical, and spiritual development of children. Physical education was a cornerstone of his pedagogy [120], as ‘the proper development of the mind depends on the proper use of the muscles and senses’ (p. 7). He viewed risky play as essential to learning: “the activities of a child are not so aimless as they seem to adults, but are the means by which [they become] acquainted with [their] world and by which [they learn] the use and limits of [their] own powers (p. 8). Thus, innovative playgrounds appealed to Washburn, who approved the first installation of a jungle gym at the North Shore Country Day School in 1920 [116]. Hinton continued to innovate his design until his death in 1923. A decade later, his wife, Carmelita Chase Hinton, another Dewey acolyte and progressive educator, founded The Putney School in Putney, Vermont.

Hinton’s use of evolutionary reasoning––an apparent first in US patent history––is an underappreciated aspect of his legacy. In 1923 [117], he argued that ‘climbing is the natural mode of locomotion which the evolutionary predecessors of the human race were designed to practice’, before concluding that it is ‘ideally suited for children’ (p. 1). These words predate the announcement, in 1924, of the Taung skull, the first-known specimen of *Australopithecus*, a hominin genus with unequivocal adaptations for climbing [121, 122]. They also came at a fraught time in American cultural history, preceding the infamous Scopes ‘Monkey’ Trial of 1925. Thus, Hinton wrote with considerable conviction and courage, but he never lived to see the prescience of his words. A century on, his views on the importance of climbing during human evolution are essentially unassailable, bolstered by decades of field research in paleoanthropology and primatology.

**Figure 2.**
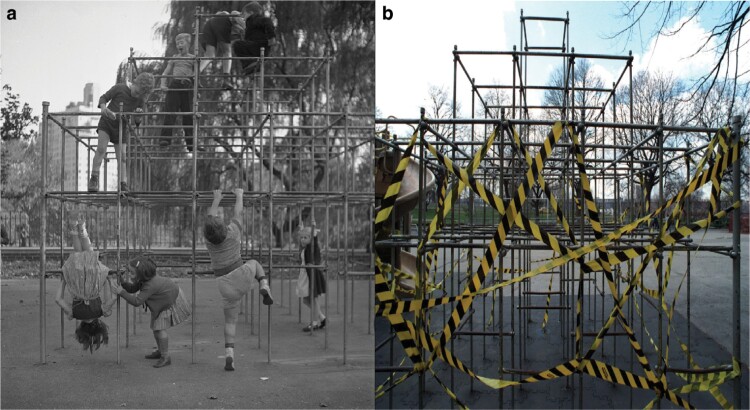
Rise and fall of Hinton’s jungle gym/monkey bars in New York City. (a) Children climbing in Central Park circa 1942. Photograph by Majory Collins, *source*: Library of Congress. (b) Closure and subsequent erasure of Hinton’s legacy at the 83rd Street Playground in Riverside Park. Photograph taken in 1996 by Steve Burman. © New York Times, reproduced with permission.

In light of these injuries, the US Consumer Product Safety Commission published a Public Playground Safety Handbook [25, 26] with height guidelines for monkey bars (described as horizontal overhead ladders). Devoid of anthropometric or kinematic data, the handbook leans heavily on the word ‘hazard’ to recommend maximum inter-rung distances of 12 inches (30.5 cm) and 15 inches (38.1 cm), with maximum heights of 60 inches (152 cm) and 84 inches (213 cm), for preschool- and school-aged children, respectively. These guidelines are not requirements, but seven states have codified them into law [4]. Compliance with these parameters has proven difficult, leading many municipalities and schools to remove monkey bars from their playgrounds. For example, the New York City Department of Parks and Recreation removed monkey bars from a majority of its 862 playgrounds during the 1980s and 90s [27] (Fig. 2). Still, a recent survey of 49 playgrounds in New Jersey, USA found that 100% of monkey bars exceeded 1.5 m and 37% exceeded 2 m in height [28]. At one primary school, the vertical height exceeded 2.5 m, which begs the question: at what height do monkey bars become a hazard?

Many public health professionals view monkey bars as unacceptably risky [29], a perspective with scant evidence. Death on playgrounds is essentially nil, with risk calculated at 0.15 per 100 000 children [16] or 1 in 30 million [30]. In other words, driving toward a playground carries a greater risk of childhood death than falls from monkey bars [30, 31]. Parents cite injury concerns for limiting thrill-seeking play on playgrounds, but such risk is low––calculated between 0.26 and 0.59 injuries per 100 000 uses [32]––and dwarfed by organized sports or gym class as causes of hospitalizations [33, 34]. Though painful, injuries resulting from falls from monkey bars rarely cause permanent harm [35]. Most playground injuries are low severity; nearly 95% of ED visitors are treated and released without further hospitalization [36]. Buckle and greenstick fractures of the distal radius or ulna can be immobilized with removable splints, wraps, or soft casts for as little as three weeks (Fig. 1a) [37]. For children under 10 years of age, some displacement (50%) in complete fractures is acceptable without referral to an orthopedist [37]. Yet, even with severe injuries, children are remarkably resilient. A recent study of pediatric admissions found that 88% and 92% of children hospitalized for severe injuries returned to baseline quality of life at 4 and 12 months post-injury, respectively [38].

Such evidence instead points to monkey bars as posing a modest risk. Yet, societal and parental tolerance of risk generally––and monkey bars more specifically––is in rapid decline across Western cultures [39]. Wyver *et al*. [30] pointed to a shift in views toward child safety––from the community (How can *we* keep *our* kids safe?) to the individual (How can *I* keep *my* kids safe?)––to explain how the subject transformed into a modern moral imperative [7, 40]. This shift in the focus of responsibility coincides with the emergence of intensive parenting––defined as over-extended, child-centered devotion of time, money and energy [41]––and the rise of ‘surplus safety’ environments [30], as exemplified by the proliferation of ‘ultra-safe’ playgrounds during the 1990s and the ensuing ‘bubble wrap’ generation [42]. Parents who ignore efforts to maximize child safety may be viewed as negligent, amplifying pressure to adhere to surplus safety, even if their own lived experiences speak to the benefits of thrill-seeking play [43]. With the weight of public consciousness so focused on the physical and reputational costs of playground injuries, we rarely consider the potential benefits, particularly from the evolutionary perspectives of biological anthropologists.

## PSYCHOLOGICAL BENEFITS OF THRILL-SEEKING PLAY

On shorter timescales, thrill-seeking play may actually mitigate childhood injuries by honing risk-perception skills [44–47]. Children as young as four have an awareness of their physical abilities and will express caution on playground equipment beyond their capabilities [2]. This argument raises the possibility that ultra-safe playgrounds are detrimental to the development of accurate risk assessment, but it is a challenging concept to test. Related to this idea is the paradoxical notion that ultra-safe playgrounds might even promote excessive risk-taking, or ‘risk compensation’. Examples include the inappropriate use of safety equipment [48, 49] and the increased allure of higher-risk settings for play behaviors (e.g. train tracks, roads, etc.) [47]. Complementing this idea is the argument that intensive parenting diminishes risk perception via negative feedback during routine supervision (e.g. ‘slow down’, ‘not so high’, and ‘be careful’), words that could instill doubt in a child’s innate judgment [46].

On longer timescales, thrill-seeking play may promote children’s mental health by enhancing self-confidence, improving coping skills and promoting resilience [50]. In their seminal article, Sandseter and Kennair [3] described the evolutionary paradox inherent in thrill-seeking play––navigating monkey bars may cause injury, but low-risk thrills also inure children to maladaptive fear levels. In other words, thrill-seeking play strengthens psychological coping mechanisms––such as diminishing an innate fear of heights––that is essential for child development. Dodd and Lester [51] extended this model by folding in a discussion of anxiety, arguing for a developmental mismatch between a child’s innate proclivity for risk-taking and the rise of ultra-safe and intensively-parented play spaces, factors that may have contributed to parallel increases in youth anxiety.

Supporting this inferred causation, Dodd *et al*. [52] used data from two online surveys of Canadian parents to demonstrate an association between thrill-seeking play and positive behavioral outcomes during the coronavirus disease 2019 pandemic. Specifically, they found that greater time spent in thrill-seeking play was associated with a reduction in internalizing problems (e.g. fears, worries, nervousness, poor relationships with peers), and greater positive affect, suggesting that thrill-seeking play mitigated some of the uncertainty caused by pandemic-era lockdowns. Another study found that children of self-reported ‘challenge parents’––parents who encourage thrill-seeking play by their children––experienced fewer anxiety symptoms [53]. This finding may explain why overprotective ‘helicopter’ parenting is linked with diminished coping skills, perfectionism and narcissism in young adults [54, 55], as well as reductions in internal locus of control [56]. The latter result has far-reaching importance; individuals with a strong internal locus of control, who believe that their own efforts matter more in the direction of their lives than external events [57], have better mental health outcomes [56].

## PHYSICAL BENEFITS OF THRILL-SEEKING PLAY

Thrill-seeking play is a frequent source of joy [45], and it is positively correlated with higher physical activity levels [58, 59]. It is practiced by children regardless of gender, although some nuance in the type of thrill-seeking play may exist [20]. In a randomized control study of 5–7 year-olds, Engelen et al. [60] found that riskier playground environments led to a 12% increase in moderate-to-vigorous physical activity relative to controls. Thrill-seeking play also promotes motor training [50], which is thought to stimulate cognitive and muscle development while fine-tuning the motor skills needed during adulthood [51]. Five-year-old children with ready access to independent thrill-seeking play had significantly better motor skills than those with fewer opportunities [61]. In another group of 5–7 year-olds, daily access to risky outdoor play (1–2 hr per day) was linked with significantly higher balance and coordination scores on a standardized motor fitness test [62]. Greater motor competency could stem from ‘self-handicapping’ [63], defined as the deliberate creation of physically challenging, moderately frightening, and unpredictable scenarios requiring locomotor versatility to overcome. The hypothesized advantage of such play is that it trains children to cope with comparable scenarios during adulthood [7].

On longer timescales, vigorous thrill-seeking play could enhance skeletal mass and strength [64]. For instance, adding 30 min of moderately intense physical activity per day can increase the bone strength of 5–11 year-olds by 3–5% [65]. Exercise-induced bone deposition occurs mainly on periosteal surfaces, providing crucial fracture resistance during aging; indeed, these material changes can be maintained throughout adolescence and even into adulthood [64]. Five-year-old children who engaged in frequent moderate-to-vigorous physical activity had higher bone mineral contents (a proxy for later gains in bone mass) at 8 (6–14% higher) and 11 years of age (4–7% higher) compared to lower physical activity groups, even after controlling for physical characteristics and current activity levels [66]. Another longitudinal study of former gymnasts found that frequent mechanical loading of the forelimbs during adolescence was linked to greater bone mass and size than non-gymnasts, with such changes detectable 4–9 years after activity cessation [67]. Likewise men who engaged in more rigorous forelimb loading during youth maintained proportionately larger and stronger humeri than control groups, even after 50 years of de-training [68].

## SELECTIVE PRESSURES ON THRILL-SEEKING PLAY

Evolutionary reasoning guided Hinton’s patent application in 1924 [Box 1], but this insight is all but forgotten in the current debate over playground safety, monkey bars and child growth and development. Our goal here is to revisit Hinton’s premise by drawing on a century of discovery in our disciplines of biological anthropology and primatology. This perspective has the potential to move debate forward in productive ways.

Climbing is essential to primate life, facilitating the acquisition of food, escape from predation, and sleep. It was practiced by our earliest ancestors, the hominins [Box 2], and it continues to hold importance for many foraging peoples [69–71]. Death from injuries related to climbing is a risk factor for all primates, but it is attributed primarily to fall impacts from extreme heights. For example, food-harvesting in some hunter-gatherers entails tree-climbing to heights up to 50 m, with fall impacts accounting for ~7% of male deaths in the Baka of the Central African Republic and ~2% in the Agta of the Philippines [71]. Chimpanzees and orangutans commonly climb to heights exceeding 20 m [72, 73], and 4–10% of chimpanzee mortality is attributed to injuries sustained from falling [74, 75]. Teleki [76] and Goodall [74] described 51 falls by Gombe chimpanzees; of these, 41% were >5 m and 25% were >10 m, with one juvenile and one adult dying subsequent to drops of 14 and 25 m, respectively. Shimada and Yano [77] described a fall of 7 m for a juvenile chimpanzee; the individual recovered, but was temporarily immobilized with head trauma. Among wild orangutans, 76% of individuals treated by veterinarians for serious injuries fell from heights of 20–50 m, with two dying of skull trauma [78]. There is also evidence of fall-induced deaths in the hominin fossil record [Box 2].

**Box 2—Paleoanthropology of climbing**

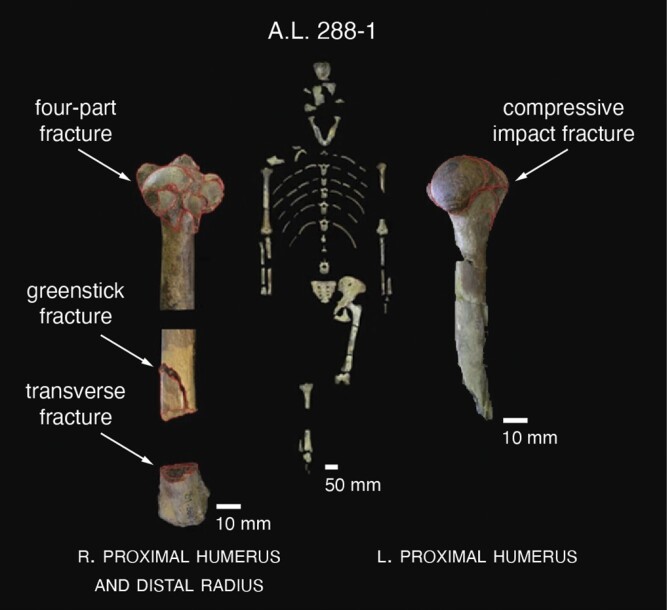

Perimortem fractures (outlined in red) of the upper extremity of A.L. 288-1 (‘Lucy’), an adult female *Australopithecus afarensis*. Photographs by J. Kappelman, reproduced with permission.

**Figure 3.**
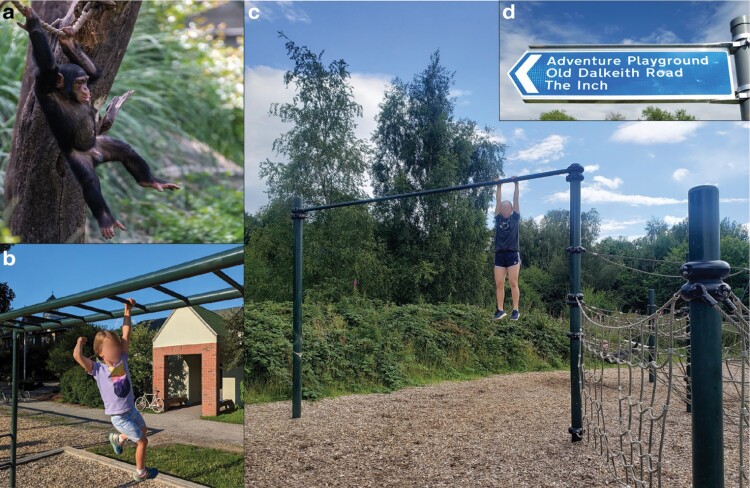
Suspensory behaviors of juvenile primates. (a) Juvenile chimpanzees spend more time climbing and swinging than adults [86], a pattern shared with humans; photograph by Eric Kilby, reproduced with permission. (b) Child arm-swinging on modern-day monkey bars with a maximum height of 2.2 m. Compare the mulched surface with those from earlier eras in Fig. 2. Location: Norwich, Vermont, USA; photograph by Z.M.T. (c) In Europe, adventure playgrounds are designed to promote thrill-seeking behavior, including potential fall heights exceeding 3 m; photograph by N.J.D. (d) Street sign highlighting the adventurous philosophy of Craigmillar Castle Park Playground, Edinburgh, Scotland; photograph by N.J.D.

Ample evidence of arboreal activity exists in the human fossil record, including a 3.3 million-year-old (Ma) infant of *Australopithecus afarensis*, the Dikika child [122]. Climbing adaptations include a gorilla-like shoulder, chimpanzee-like fingers, and a flexible foot [123, 124]. The infant died at ~3 years of age, and it is tempting to imagine its propensity for thrill-seeking play in the treetops of Pliocene Africa; indeed, its age falls squarely in the age-range of maximum arborealism and suspension among chimpanzees (Fig. 3a) [86, 89].

Still, some hominin fossils hint at mortality from falls. For instance, Kappelman et al. [125] described bone fractures in the skeleton of ‘Lucy’––an adult female *A. afarensis* dated to 3.2 Ma––and attributed them to a fall of ~13 m. The authors identified perimortem fractures consistent with vertical deceleration impacts along both forelimbs, including a four-part fracture of the proximal head of the right humerus, a greenstick (or hinge) and spiral fracture on the lateral surface of the right humeral midshaft, a compressive impact fracture on the proximal head of left humerus, and a transverse break of the right distal radius. Such fractures suggest a high-energy impact scenario with outstretched arms. It is interesting––and perhaps not coincidental––that the greenstick fracture is comparable to those commonly found in pediatric patients that fall from playground equipment (Fig. 1a) [37]. In another example, L’Abbé et al. [126] attributed a distal ulnar bending fracture in the skeleton of MH2––an adult female *A. sediba* dated to ~2.0 Ma––to a fall. Like those of Lucy, the fracture indicates an axially-placed load on the forelimb, suggesting an attempt to break its fall with outstretched arms.

These heights and the corresponding mortality risks do not apply to most playgrounds today. A better point of comparison revolves around the risk of skeletal fracture. Healed fractures are found in the long bones of all great ape species, suggesting that nonfatal falls are somewhat frequent [79]. For example, the frequency of healed bone fractures (typically forelimbs) ranges from 21% to 36% of individuals in populations of wild chimpanzees [79, 80]. Comparable rates are evident among wild gorillas (20%), gibbons (36%), and orangutans (61%) [81, 82], as well as those human populations that climb trees often [71]. In one example from Papua New Guinea, tree falls accounted for 27% of hospital admissions, most of which involved fractures of the distal radius [83]. Falls from mango trees accounted for 16% of admissions for skeletal fractures at a hospital in Fiji, with 69% being pediatric inpatients and 56% of fractures occurring in the forelimb [84]. Likewise, falls from coconut trees resulted in a large number of admissions at a rural hospital in the Solomon Islands, with 57% of falls resulting in fracture; and it is telling that 40% of admittees were 10–14 years old [85]. Thus, skeletal fracture and recovery is a relatively common experience in the life history of most apes, including humans.

It follows that natural selection should operate strongly on juvenile primates to master the art of climbing; indeed, climbing and swinging are more common among younger age classes than any other ontogenetic stage (Fig. 3) [87, 88]. Among chimpanzees, infants (0–5 yrs) and juveniles (5–10 yrs) spend 15% and 27% more time climbing and arm-swinging than adults (+ 20 yrs), respectively [86]. This difference exists because infants and juveniles allocate more time (~40%) to arboreal activities [89]. Not only are juveniles more apt to climb than adults but they also exhibit a far greater diversity of movements and postures [87]. Exemplifying this point, juvenile langurs have 65% more distinct positional behaviors (both postures and movements) and 92% more distinct suspensory positional behaviors than adults [90]. Such locomotor experimentation is a crucial part of juvenile development, as it enables the fine-tuning of motor skills needed during adulthood [88]. Tellingly, infant chimpanzees that engage in more social play (a category that included locomotor play) tend to reach social (e.g. first non-maternal groom) and motor (e.g. first independent travel) milestones at earlier ages [91]. Thus, play-climbing represents a form of practice: one that promotes an awareness of branch material properties––an important consideration when navigating arboreal environments [92, 93]––and the mechanical loading caused by one’s own mass. It is best to practice these perceptual skills at a size that minimizes the force of impact and risk of injury. Yet, mounting morphological and physiological evidence points to juvenile skeletons being resistant to the mechanical challenges and impacts that occur during thrill-seeking play.

Juvenile primates have underdeveloped bodies and weaker muscles than adults, yet they move in riskier ways; so it stands to reason that natural selection has acted on their skeletons to improve locomotor efficiency and safety [94]. In terms of efficiency, juvenile primates possess higher effective mechanical advantage of their limb muscles than adults, meaning juveniles apply relatively less muscular force to produce the same output forces for a given posture [94]. For example, juvenile capuchins enjoy a greater anatomical mechanical advantage (AMA) of the biceps brachii and triceps brachii muscles, increasing the efficiency of suspensory behaviors [95]. Likewise, juveniles have better grasping abilities than adults because they have disproportionately large digits [94, 96]. For example, the relatively wide phalanges of juvenile baboons enable disproportionate pull strengths, exceeding adult values by 200% [97].

Juvenile bones are also relatively fracture-resistant. For example, the juvenile humerus and femur have disproportionately greater bending strengths than those of adults [98], in part because mineralization is incomplete. In short, juvenile bones are structurally tougher and more elastic, factors that absorb energy and minimize fracture severity [94]. Capuchin monkeys have the highest bone safety factors (i.e. the strength of their bones relative to predicted loads) at the onset of juvenilization, when the risk of falling is greatest [98]. Similarly, the humeri of chimpanzee infants (0–5 yrs) are relatively more resistant to bending and torsion than those of older individuals [89], a pattern shared with human children of similar ages [99]. These traits could be linked to the greater head-to-body mass ratio of juveniles, which moves their center of mass cranially and favors use of the hands to minimize the impacts of falls [100]. Using outstretched hands to mitigate head trauma during falls could explain the evolution of disproportionate forelimb strengths at the juvenile stage of development [98]; so, it is telling that many of the buckle and greenstick fractures to the radii and ulnae of children are the result of hands-first impacts (Fig. 1a) [37].

## OUR VIEWS AS BIOLOGICAL ANTHROPOLOGISTS

Like Hinton [Box 1], we view play-climbing as a legacy of primate evolution that promotes child health and well-being. But this perspective is often overshadowed by the arguments of public health professionals and policymakers, who tend to problematize forelimb fractures as a costly and preventable playground injury. The tension between these perspectives is evident in the diverging play-climbing standards of several countries. In Australia, the maximum acceptable ‘free height of fall’ for climbing equipment was *increased* from 2.5 to 3.0 m in 2014 (standard AS 4684-2014), putting it into alignment with an earlier European standard (EN 1176-2008) (Fig. 3c) [101]. Conversely, many clinicians have argued for free-fall heights <2.0 m, with some favoring reductions to ≤1.5 m [21–24]. Both perspectives are rooted in statistical data, giving the impression of evidenced-based policymaking, but there is a problem: there is scant empirical data on *how* children interact with climbing equipment. To fill this void, it is useful to consider the tools of primatologists, researchers adept at quantifying limb movements during climbing [102, 103] or applying deep learning methods to track postures [104]. Such data would inform our understanding of how playground equipment shapes the childhood propensity for thrill-seeking play, moving us closer to human-centered design principles.

**Figure 4.**
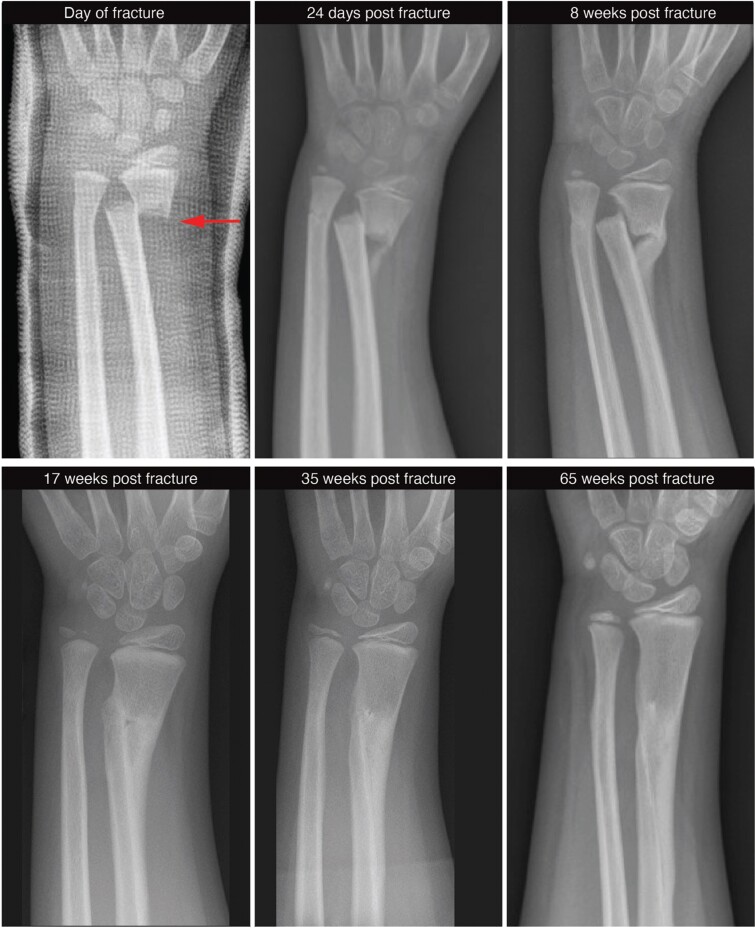
A seven-year-old boy climbing on monkey bars suffered a fall, resulting in a displaced fracture of the right distal radius (arrow). Despite the severity, the attending orthopedic surgeon opted for nonoperative treatment. Serial radiographs over the next 15 months illustrate the natural remodeling of bone. Case history and images courtesy of James Gamble, Stanford University School of Medicine.

When faced with forearm shaft fractures, pediatric orthopedists have turned increasingly toward nonoperative treatments, even in severe cases [105–107]. This trend speaks to the outstanding resilience of juvenile forelimbs (Fig. 4), and it suggests that a wide safety margin is baked into the juvenile life stage, a premise that may inform enduring questions related to human life history evolution. Juvenile growth among primates, especially humans, is exceedingly slow [108], but the selective advantages of prolonging this life history stage are debated. It could mitigate against periods of low food availability [108, 109] or enable the development of advanced foraging and social skills [110–112]. Another possibility is that it is essential for developing motor competence, an idea linked to the evolution of play behaviors [63, 113], but not extended juvenility. It follows that arborealism is a strong predictor of juvenile duration across primates; and that the evolution of the hominin foot and ankle––traits that may have increased the risk of falling during arboreal activities [114]––was a major selective force on human life history evolution. In short, the selective advantages of motor competency in an arboreal milieu may have contributed to the evolution of prolonged juvenility across primates, and particularly hominins, a hypothesis that invites future research.

## SWINGING FORWARD

Ensuring the safety of children is a justifiable concern, and we understand societal apprehension over the relatively high rates of fracture associated with monkey bars. What is less understood are the long-term physical and mental health costs of reducing or eliminating monkey bars or other catalysts of thrill-seeking play. In debates over the costs and benefits of such play, it is practical to consider the regularity of forelimb fractures across the arc of primate evolution, a perspective that begins to contextualize the injury with the adaptive importance of thrill-seeking play during the juvenile life stage of primates, especially humans. We believe the enduring appeal of monkey bars today is a testament to this evolutionary legacy, and that we must find ways to avoid extreme injuries while also letting children face developmentally appropriate risks.

A century ago, Hinton invented monkey bars to encourage primate-inspired play. Over the next 100 years, we may consider taking the next step of encouraging thrill-seeking play with natural materials in less-developed settings [115]. For instance, outdoor ‘adventure’ playgrounds have emerged in recent years across North America [7]. Already common in Europe, adventure playgrounds offer standard challenges (e.g. great heights; Figure 3c), as well as loose elements that encourage interactive and constructive play; e.g. recycled junk, nature-based materials, and access to fire and water [7]. Adventure playgrounds exemplify the spirit and intent of Hinton’s original desire for innovative playground equipment, and we hope that future discussions of thrill-seeking play will acknowledge the potential benefits while continuing to draw inspiration from our evolutionary and arboreal legacies. 
